# Structure-activity exploration of a small-molecule allosteric inhibitor of T790M/L858R double mutant EGFR

**DOI:** 10.1080/14756366.2022.2145284

**Published:** 2022-11-13

**Authors:** Francesca Foschi, Annachiara Tinivella, Valentina Crippa, Luca Pinzi, Luca Mologni, Daniele Passarella, Giulio Rastelli

**Affiliations:** a Department of Life Sciences, University of Modena and Reggio Emilia, Modena, Italy; b Department of Chemistry, University of Milano, Milano, Italy; c School of Medicine and Surgery, University of Milano-Bicocca, Monza, Italy

**Keywords:** EGFR inhibitors, allosteric inhibitors, anticancer drugs, drug design

## Abstract

EGFR is a protein kinase whose aberrant activity is frequently involved in the development of non-small lung cancer (NSCLC) drug resistant forms. The allosteric inhibition of this enzyme is currently one among the most attractive approaches to design and develop anticancer drugs. In a previous study, we reported the identification of a hit compound acting as type III allosteric inhibitor of the L858R/T790M double mutant EGFR. Herein, we report the design, synthesis and *in vitro* testing of a series of analogues of the previously identified hit with the aim of exploring the structure-activity relationships (SAR) around this scaffold. The performed analyses allowed us to identify two compounds **15** and **18** showing improved inhibition of double mutant EGFR with respect to the original hit, as well as interesting antiproliferative activity against H1975 NSCLC cancer cells expressing double mutant EGFR. The newly discovered compounds represent promising starting points for further hit-to-lead optimisation.

## GRAPHICAL ABSTRACT

**Figure:**
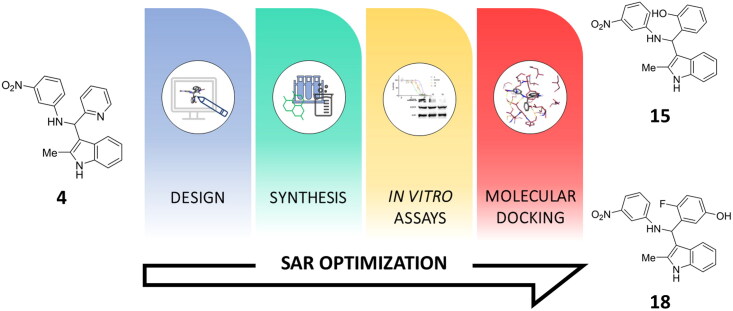

## Introduction

The Epidermal Growth Factor Receptor (EGFR) is a protein tyrosine kinase of pivotal importance for the development of anticancer drugs^1^. Its function depends on the strictly regulated shift between active and inactive conformations, which can be distinguished by the different positions of key structural elements such as the DFG-motif and the αC helix^2–5^. Primary mutations in the EGFR sequence such as the L858R are known to be involved in tumour development and drug resistance, especially in non-small-cell lung cancer (NSCLC)^6^^,^^7^, accounting for more than 80% of highly fatal lung cancers^8^^,^^9^. Indeed, L858R promotes the active conformation of the αC helix, destabilising the inactive state^2^^,^^3^. Although a number of ATP-competitive inhibitors of EGFR has been approved by the Food and Drug Administration (FDA) so far, the onset of secondary mutations, such as the T790M at the gatekeeper makes most of these drugs ineffective as it alters enzymatic activity and drug binding, by increasing affinity of the protein for ATP^10^. Affinity for ATP, however, reverts to levels comparable to wild-type EFGR in the concurrent presence of activating mutations such as L858R, which reduces the effectiveness of first- and second-generation ATP-competitive EGFR inhibitors. Likewise, the C797S mutation abrogates the activity of several irreversible inhibitors, as it removes the covalent reactive cysteine^11^. For these reasons, the discovery of allosteric inhibitors of EGFR, and, in particular, of the more clinically relevant dru g resistant forms, has become of great interest as a possible way for circumventing drug resistance^12^^,^^13^.

**Figure 1.**
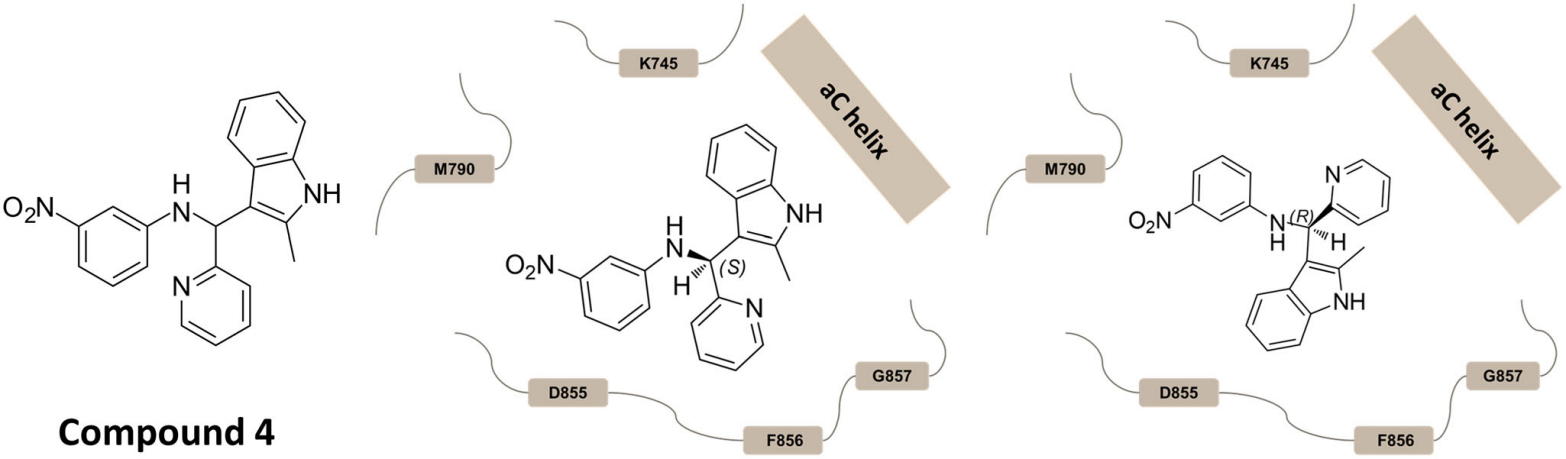
Chemical structure of the hit compound **4**, and the binding modes predicted on EGFR by docking^14^.

In a recent study, a high throughput docking screening performed on a set of commercially available molecules led to the identification of a hit candidate (compound **4** in Figure 1) acting as type III allosteric inhibitor of T790M/L858R double mutant EGFR, with an EC_50_ of 6.2 ± 0.5 μM^14^. Of note, the compound showed also anti-proliferative activity against three NSCLC cell lines exhibiting different EGFR status, the observed IC_50_ values being 14.5 ± 0.3 μM (*wt* EGFR H1299 cell line), 19 ± 2 μM (mutant EGFR H1650 cell line), and 24 ± 2 μM (double mutant H1975 cell line)^14^.

Following our interest in developing allosteric inhibitors of protein kinases^14–17^, in this work, we sought to explore the structure-activity relationships (SAR) around this hit compound by designing, synthesising and *in vitro* testing a library of seventeen analogues bearing structural modifications at both the phenyl, pyridine and indole rings. Two of the designed compounds were found to be significantly more active than the parent hit against double mutant EGFR. The two compounds were further characterised for their anti-proliferative activity against the H1975 NSCLC cell line expressing the double mutant.

## Results

### Molecular design

Starting from the structure of the hit compound **4**, we explored chemical modifications in all three aromatic rings (Table 1). These modifications were inspired by the docking complexes of compound **4** in complex with mutant EGFR^14^.

**Table 1.** Chemical structures of the synthesised compounds.

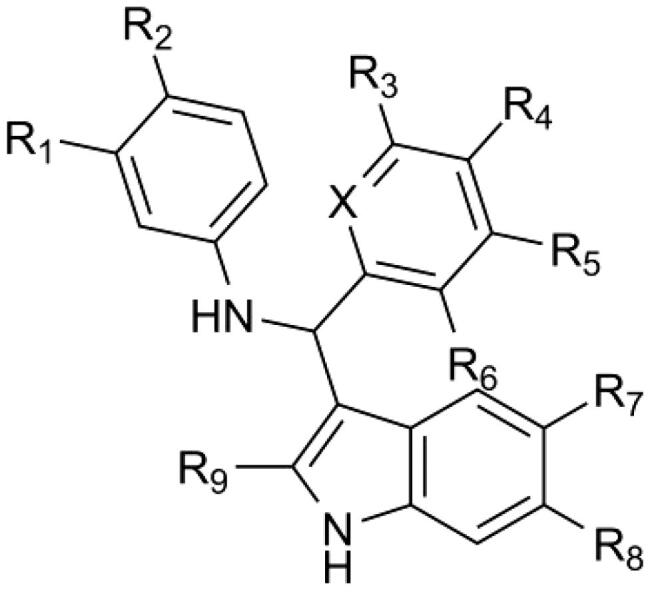										
Compound	X	R1	R2	R3	R4	R5	R6	R7	R8	R9
**4**	N	NO_2_	H	H	H	H	H	H	H	CH_3_
**5**	N	H	NO_2_	H	H	H	H	H	H	CH_3_
**6**	N	OH	H	H	H	H	H	H	H	CH_3_
**7**	N	NO_2_	H	H	H	H	H	F	H	CH_3_
**8**	N	NO_2_	H	H	H	H	H	H	H	H
**9**	N	NO_2_	H	H	H	H	H	H	COOMe	H
**10**	N	NO_2_	H	H	H	H	H	COOMe	H	H
**11**	N	NO_2_	H	F	H	H	H	H	H	CH_3_
**12**	N	NO_2_	H	H	H	H	OH	H	H	CH_3_
**13**	CH	NO_2_	H	H	H	H	H	H	H	CH_3_
**14**	CH	NO_2_	H	F	H	H	OMe	H	H	CH_3_
**15**	CH	NO_2_	H	H	H	H	OH	H	H	CH_3_
**16**	CH	NO_2_	H	OH	H	H	H	H	H	CH_3_
**17**	CH	NO_2_	H	F	H	H	OH	H	H	CH_3_
**18**	CH	NO_2_	H	OH	H	H	F	H	H	CH_3_
**19**	CH	NO_2_	H	OH	H	H	H	F	H	CH_3_
**20**	CH	NO_2_	H	OH	H	H	F	F	H	CH_3_

Firstly, the nitro group in the meta position was moved to the para position (**5**) or substituted with a hydroxyl group (**6**). The indole moiety in **4** was then substituted with a 5-fluorine atom, providing compound **7**. Removal of the 2-methyl group of the indole ring provided **8**. The latter compound was further substituted with a methyl ester residue at the positions 6 (**9**) or 5 (**10**). As for modifications of the pyridine ring, an *m*-fluorine atom or a *o*-hydroxyl group were added, resulting in compound **11** or **12,** respectively. Attempts to introduce an additional fluorine atom on compound **12** failed. Then, the nitrogen atom of the pyridine residue was removed (**13**) and the resulting phenyl ring was substituted with an *o*-hydroxyl (**15**) or a *m*-hydroxyl group (**16**). Synthesis of the *o*-hydroxyl-*m*-fluoro derivative afforded compound **17**. Replacement of the hydroxyl group of compound **17** with a methoxy group led to compound **14**. Likewise, synthesis of the *o*-fluoro-*m*-hydroxyl derivative afforded compounds **18** and **20**.

### Chemical synthesis

**Scheme 1.**
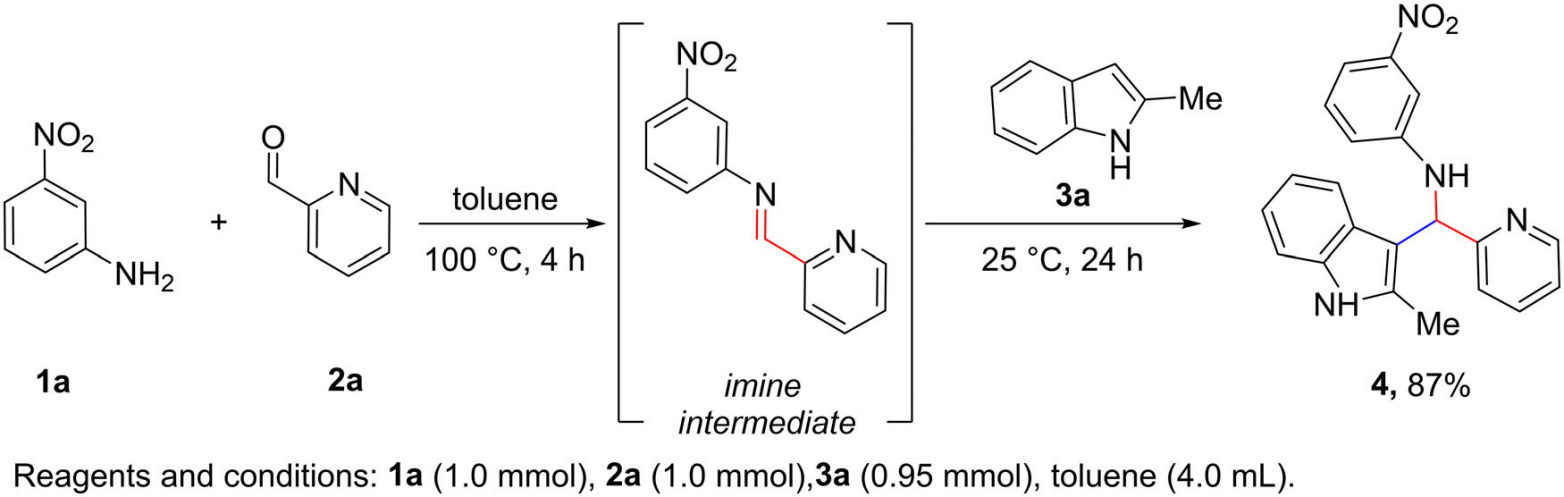
Synthesis of hit compound **4**.

The model compound of our studies (**4**, Scheme 1) was prepared via an aza Friedel-Craft (AFC) one pot process. As shown in Scheme 1, the synthesis of **4** involves an *aniline* derivative (**1a**, meta nitro aniline), an *aromatic aldehyde* (**2a**, picolinaldehyde) and an *indole ring* (**3a**, 2-methylindole): these are the three structural units object of our investigation. Furthermore, the reaction proceeds through condensation of **1a** and **2a** followed by the ACF reaction of indole **3a** and the imine intermediate avoiding the use of any catalyst^18^^,^^19^.

**Scheme 2.**
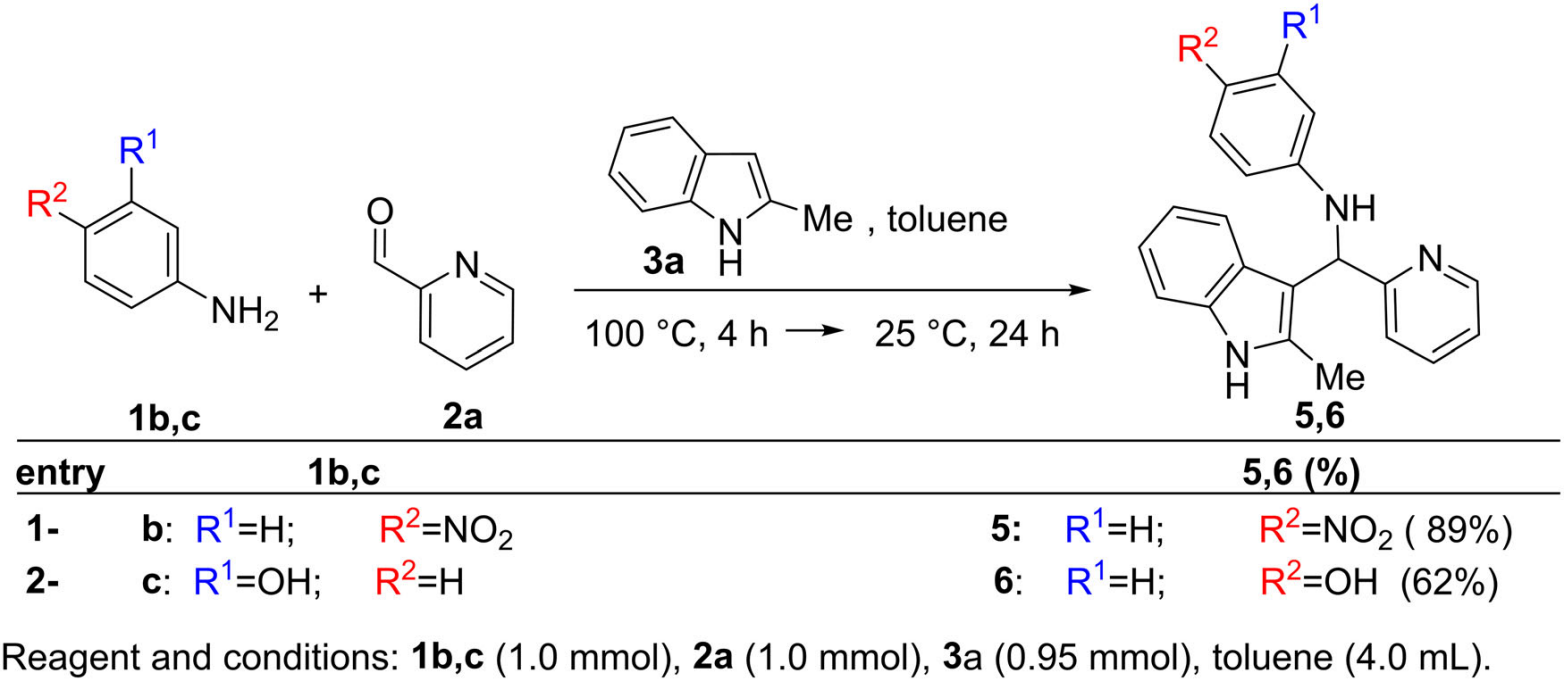
Modification at the *aromatic aniline-ring*: synthesis of **5** and **6**.

**Scheme 3.**
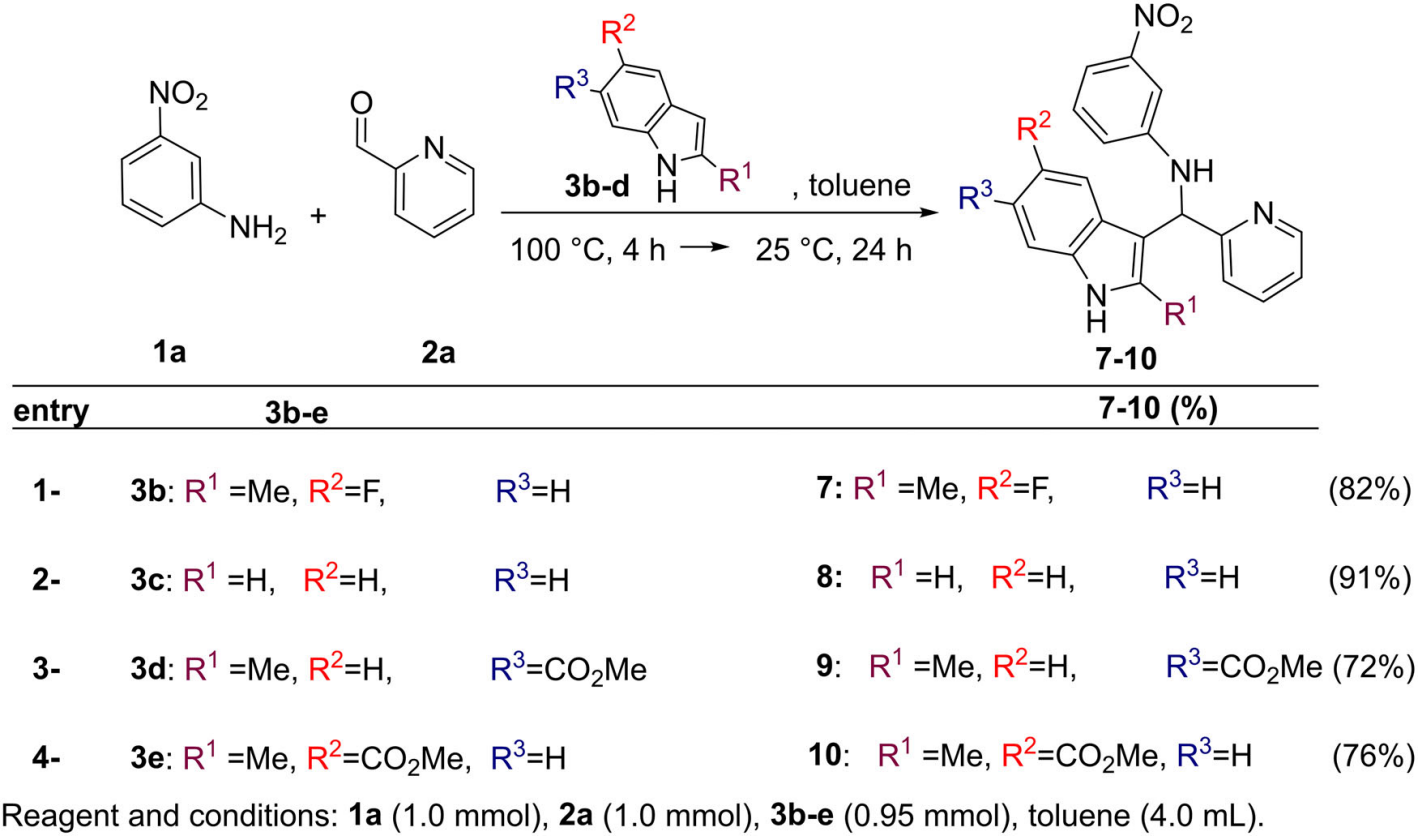
Modification at the *indole residue*: synthesis of **7–10**.

**Scheme 4.**
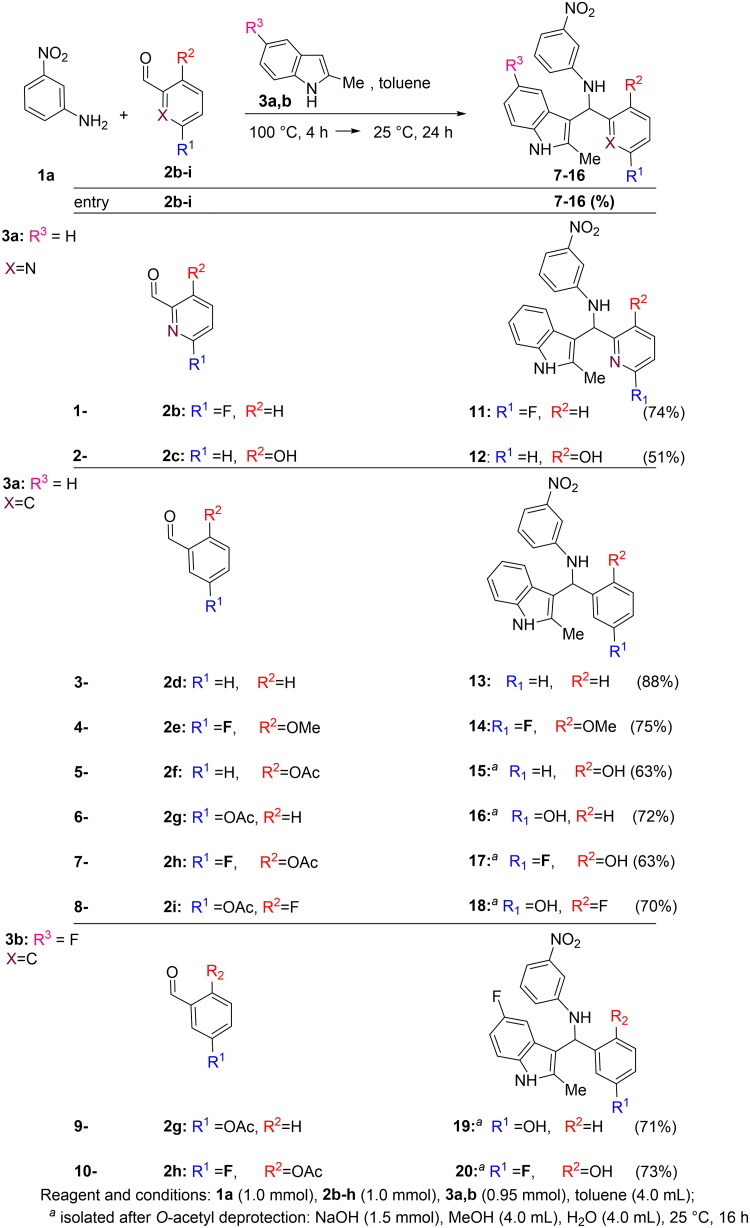
Synthesis of inhibitors **11–20**.

The generality of the multicomponent ACF process was tested on a range of substituted anilines **1**, indoles **3** and aldehydes **2** (Schemes 2–4). Furthermore, good yields of inhibitors **5,6** were reached when aldehyde **2a** and indole **3a** were reacted with anilines **1b,c** bearing functional groups whit different electronic properties (Scheme 2). A similar behaviour was observed when indoles **3b-e** were employed in the presence of aniline **1a** and aldehyde **2a** (synthesis of **7–10**, Scheme 3). Also the use of picolinaldehydes **2b,c** -bearing respectively a fluorine and a hydroxy residue- or benzaldeyde **2d** or 2-methoxy, 6-Fluoro benzaldehyde **2e** did not affect the reaction outcome (synthesis of inhibitors **11–14**, Scheme 3). On the other hand, the modulation of the electronic properties of hydroxy aldehyde **2f**,**i** was crucial to promote the reaction pathway (synthesis of **15–20**). Thus, the OH residue in **2f**,**i** was *O*-acetylated prior to the ACF reaction^20^. In turn, the final inhibitors **15–20** were isolated after cleavage of the acetyl moiety under basic conditions^21^.

### Biological evaluation

**Figure 2.**
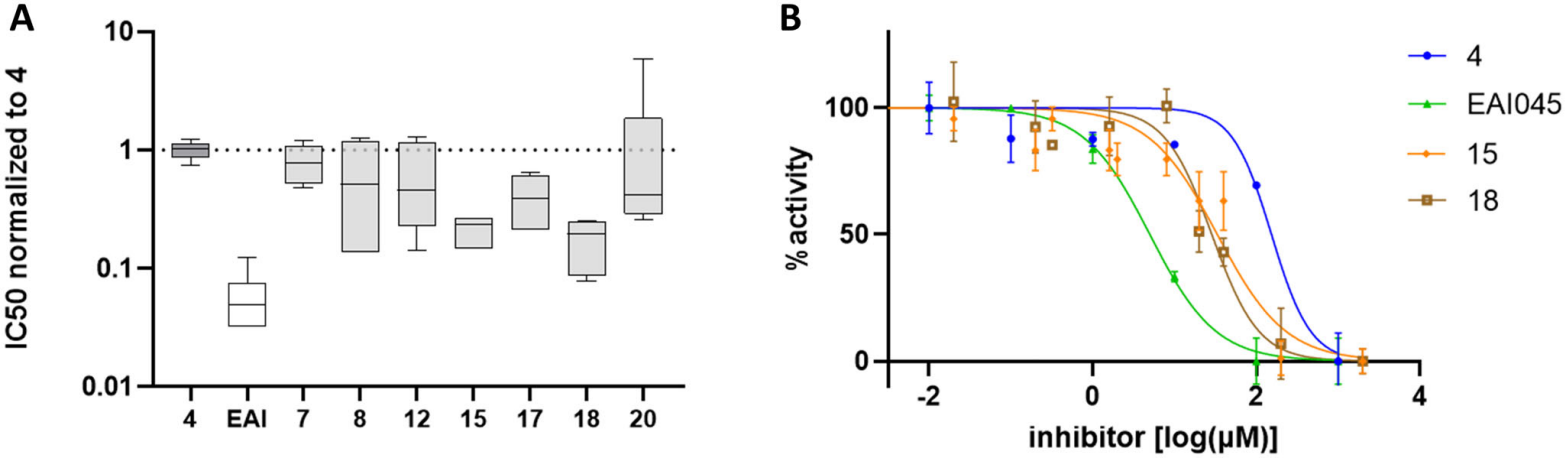
Activity of compounds in kinase assays. (A) Box and whiskers plot (min to max) of normalised IC_50_ values, relative to compound **4**, from five independent experiments. (B) Dose-response curves of the indicated compounds from one representative experiment.

The synthesised compounds were tested *in vitro* by non-radioactive kinase assay, using compounds **4** and **EAI045** as references (Table S1). As shown in Figure 2, a number of compounds showed comparable or better activity than **4**. In particular, compounds **15** and **18** showed significantly improved activity with respect to **4** (*p* < 0.0001). All other tested compounds were inactive on mutant EGFR (IC_50_ > 200 µM).

**Figure 3.**
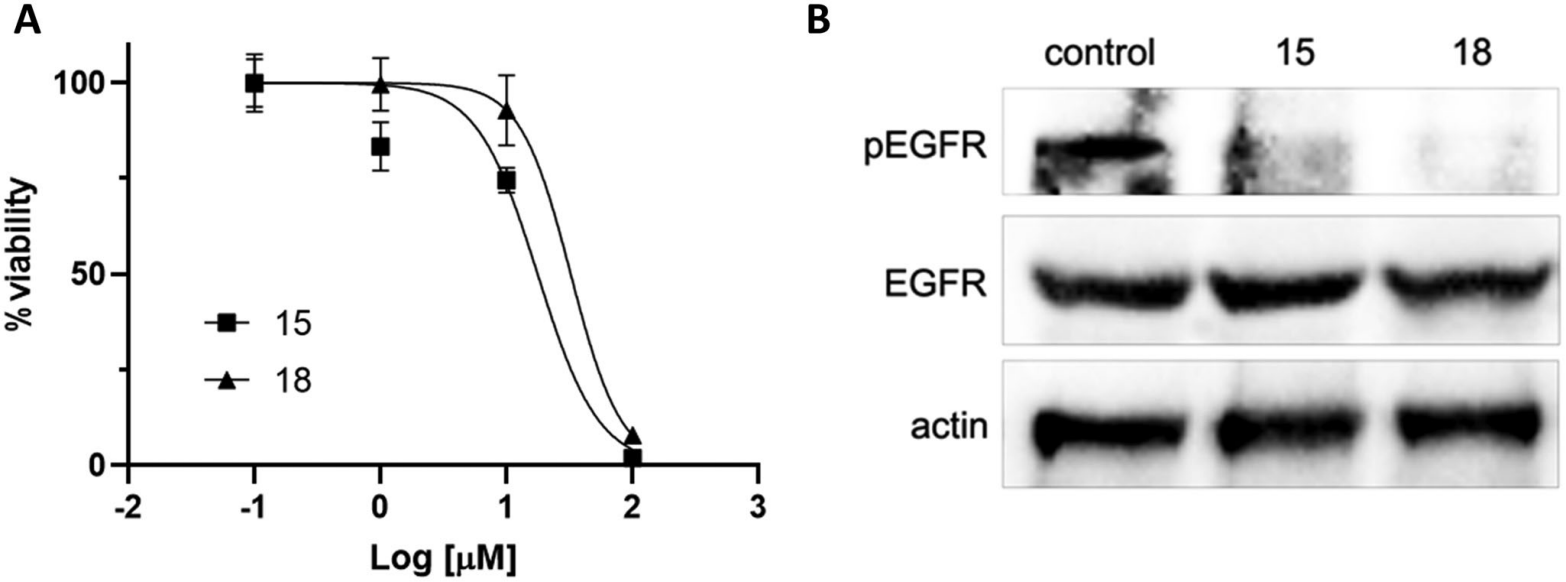
Activity of compounds **15** and **18** in H1975 cells. (A) Cell viability was measured by MTS assay. (B) EGFR phosphorylation was assessed by Western blot after 8-h incubation with the compounds (10 µM). Total EGFR and actin are shown for loading control.

Compounds **15** and **18** were then tested in cell proliferation assays on the H1975 lung cancer cell line expressing the double mutant L858R/T790M EGFR. Both compounds inhibited cell proliferation and EGFR phosphorylation at micromolar concentrations (Figure 3).

### Molecular docking

**Figure 4.**
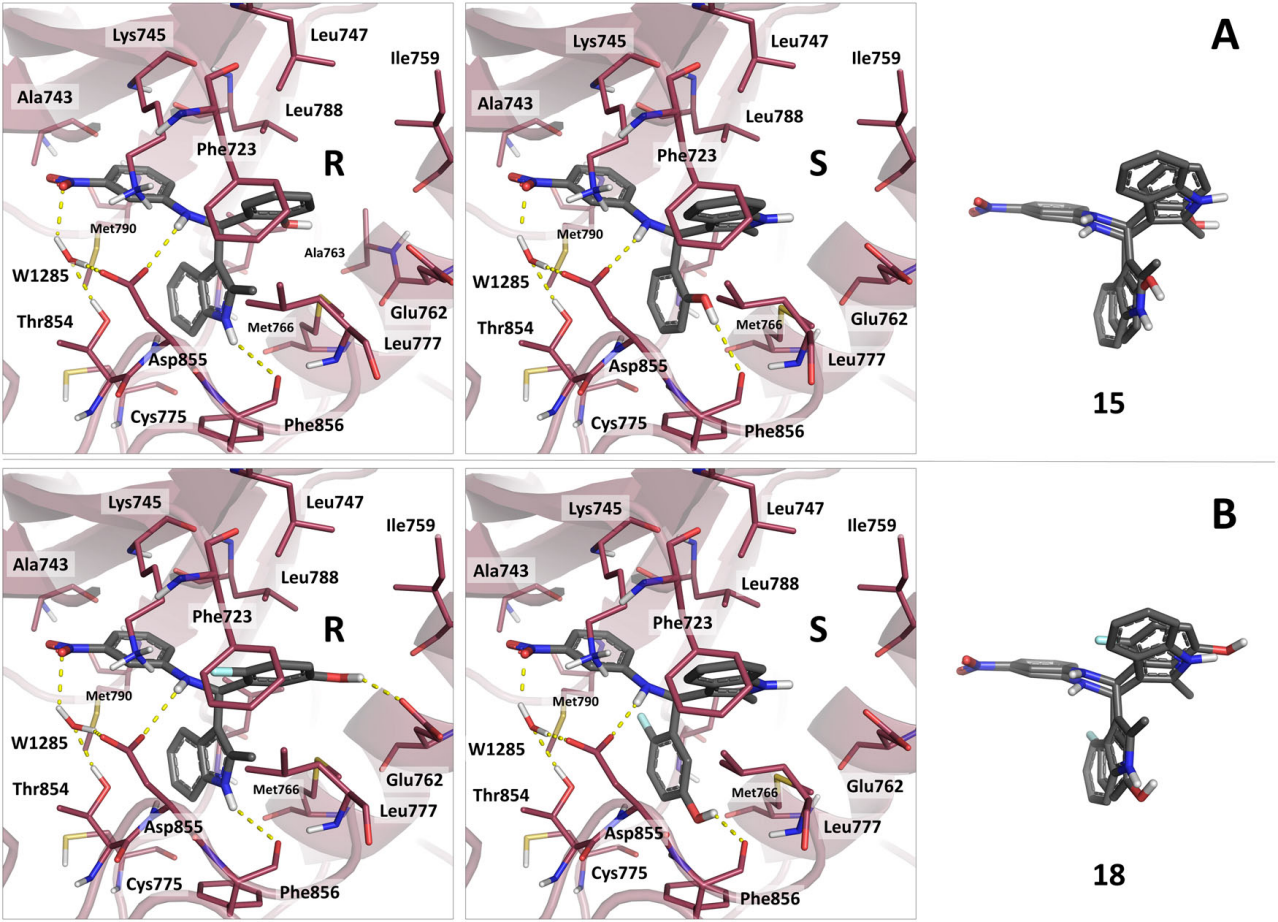
Binding mode predicted for compounds **15** and **18** by docking. (A) reports the binding mode predicted for the R and S stereoisomers of compound **15**. (B) reports the binding mode predicted for the R and S stereoisomers of compound **18**. The superimposition of the R and S stereoisomers of **15** and **18** in their predicted binding mode is also reported. As can be observed, the stereoisomers of the compounds show similar pattern of interactions, with good superimposition of their aromatic and h-bond donating groups.

Docking calculations of both the stereoisomers of the best compounds **15** and **18** were firstly performed with Glide ^22^, starting from the crystal structure of T790M EGFR in complex with EAI001 (PDB code: 5D41)^12^. In the predicted complexes, the nitrophenylamine moiety of both the compound stereoisomers is involved in a well-defined network of interactions, while the indole and hydroxyphenyl groups explored different orientations within the type III allosteric pocket. In particular, the nitro group of **15** and **18** hydrogen bonds with a water, the importance of this molecule for the binding of type III allosteric ligands to mutant T790M EGFR being previously assessed^23^. In turn, the water molecule hydrogen bonds with the side chain of Thr854 and Asp855 (Figure 4), the latter residue interacting with the amine group of the investigated compounds. The phenyl ring was predicted to establish hydrophobic interactions with the side chain of Ala743, Leu788 and Met790. The hydroxyphenyl and indole moieties of the R and S stereoisomers of compounds **15** and **18** were predicted to accommodate differently within the allosteric pocket of EGFR, albeit their superimposition revealed overlapping structural features and similar pattern of interactions with protein residues (Figure 4). In particular, the hydroxyphenyl moiety of **15S** and **18S** was predicted to accommodate near the side chain of Phe856 and Met766, the phenyl ring establishing favourable hydrophobic interactions, and the hydroxyl group hydrogen bonding to the backbone carbonyl of Phe856, similarly to what previously observed (Figure 4)^14^. In the R stereochemistry, the latter interaction is performed by the amino group of the indole instead of the hydroxyphenyl. In the **15S** and **18S** complexes, the indole portion of the compounds binds in proximity to Leu747 and Ile759, the amino group hydrogen bonds with the side chain of Glu766 (Figure 4). Besides, this interaction is mapped by the hydroxyl groups at the *ortho* and *meta* positions in the **15R** and **18R** complexes (Figure 4), respectively. The introduction at these positions of functional groups unable to establish h-bond interactions resulted in lack of activity (*e.g.* compounds **13** and **14**), suggesting that these hydrogen bonds are required for activity. Substitutions with a fluorine in the phenyl moiety are tolerated, when a hydroxyl group is present at the *ortho* and *meta* positions (*e.g.* compounds **17**, **18** and **20**) (Figure 2).

To investigate the effect of protein flexibility on the predicted docking poses ^24^, we also performed *Induced Fit Docking* (IFD) ^25^^,^^26^ calculations. Remarkably, *IFD* provided results similar to those obtained by docking with Glide. Altogether, the results of these analyses confirmed that the two stereoisomers of these compounds present good complementarity with the allosteric site of mutant EGFR. The additional hydrogen bonds formed by **15** and **18** may explain their higher activity. The 3D coordinates of the complexes of compounds **15** and **18** with double mutant EGFR are available upon request to the authors.

## Conclusions

In this study, a series of compounds were designed, synthesised, and *in vitro* tested to explore the SAR of a class of previously discovered type III allosteric inhibitors of double mutant EGFR^14^. The performed studies allowed to identify two derivatives with significantly higher inhibitory activity with respect to the starting hit compound **4**. The best compounds identified from the *in vitro* assays on EGFR (*i.e.* **15** and **18**) were also tested for their antiproliferative activity towards the H1975 NSCLC cancer cell lines expressing the double mutant, obtaining satisfactory results. Docking analyses were finally performed by means of Glide and the Induced Fit Protocol to help explaining their higher activity and rationalise the SAR of the investigated compounds. The results of this study allowed to highlight the importance of hydrogen-bonding groups at key positions of the investigated scaffold, providing interesting structural clues for further hit to lead optimisation in the allosteric pocket. Despite remarkable on-target biological activity, these hits still display off-target toxicity in EGFR-non-dependent cells (Figure S1), indicating the need for further refinement. However, the compounds are *small-in-size* and present a rather low decorated scaffold, which represent key assets for further medicinal chemistry optimisation, especially in the relatively uncharted context of type III allosteric inhibitors.

## Supplementary Material

Supplemental Material
