# Supplementary material for: Structure-activity exploration of a small-molecule allosteric inhibitor of T790M/L858R double mutant EGFR
Source: J Enzyme Inhib Med Chem. 2022 Nov 13;38(1):239–45. doi: 10.1080/14756366.2022.2145284 (PMC9665079; doi:10.1080/14756366.2022.2145284)
Supplement: Supplemental Material [file IENZ_A_2145284_SM3123.pdf]

SUPPORTING INFORMATION

Structure-activity exploration of a small-molecule allosteric inhibitor of T790M/L858R double mutant EGFR.

Francesca Foschi,[a,b,♪] Annachiara Tinivella,[a] Valentina Crippa,[c] Luca Pinzi,[a] Luca Mologni,[c] Daniele Passarella,[b] Giulio Rastelli [a*]

[a]*Department of Life Sciences, University of Modena and Reggio Emilia. Via Campi 103, 41125 Modena, Italy;* [b]*Department of Chemistry, University of Milano. Via Golgi 19, 20133 Milano, Italy;* [c] *School of Medicine and Surgery, University of Milano-Bicocca, via Cadore 48, 20900 Monza, Italy*

Corresponding author

* Prof. Giulio Rastelli

Department of Life Sciences

University of Modena and Reggio Emilia.

Via Campi 103, 41125 Modena, Italy.

Phone: +39 0592058564; E-mail: giulio.rastelli@unimore.it

♪ *Present address: Dipartimento di Scienza e Alta Tecnologia, Università dell'Insubria, via Valleggio 11, 22100 Como*

Table of Contents

Methods

Chemical synthesis ………………………………………………………………………… S3

Biological evaluation ……………………………………………………………………… S4

Molecular docking analyses ……………………………………………………………… S11

Figures

Figure S1…………..……………………………………………………………………..… S12

Tables

Table S1…………….………………………………………………………………………… S13

REFERENCES…………..……………………………………………………………….... S14

METHODS

Chemical synthesis

All available chemicals and solvents were purchased from commercial sources and used without any further purification. Thin layer chromatography (TLC) was performed using 0.25 mm silica gel precoated plates Si 60-F254 (Merck, Darmstadt, Germany) visualized by UV-254 light and CAM staining. Purification by flash column chromatography (FCC) was conducted by using silica gel Si 60, 230-400 mesh, 0.040-0.063 mm (Merck). ^{1}H and ^{13}C NMR spectra were recorded on a Bruker Advance 400 (400 and 101 MHz, respectively) or Bruker Advance 300 (300 and 75 MHz , respectively); chemical shifts are indicated in parts per million downfield from $SiMe_4$, using the residual proton (acetone = 2.05 ppm, DMSO= 2.54 ppm, MeOH= 3.33 ppm, CH_2Cl_2= 5.31 ppm, CH_3Cl= 7.26 ppm) and carbon (acetone = 29.8 and 206.3 ppm, DMSO= 39.9 ppm, MeOH= 49.0 ppm,CD_2Cl_2 = 53.5 ppm, CD_3Cl = 77.0 ppm) solvent resonances as internal reference. Protons and carbon assignments were achieved by ^{13}C-APT, ^{1}H-^{1}H COSY, and ^{1}H-^{13}C heteronuclear correlation experiments. Coupling constants values *J* are given in Hz. FTIR spectra were recorded on a Tensor 27 (ATR Diamond) Bruker infrared spectrophotometer and are reported in frequency of absorption (cm^{-1}).

<u>*1. Synthesis*</u>

General procedure for the one-pot ACF reaction

A mixture of aldehyde **2a-i** (1.0 mmol) and aniline **1a-c** (0.95 mmol) in dry toluene (4.0 mL) was stirred at 80 ° C for 4 h. Then, the reaction was cooled to 25 ° C and indole **3a-e** (0.95 mmol) was added. The resulting solution was stirred at 25 ° C for 24 hours, diluted with ethyl acetate (10 mL), washed with saturated $NaHCO_3$ solution (3×10 mL), and brine (10 mL), dried over NA_2SO_4, and filtered. After evaporation of the solvent under vacuum (RV), the crude was purified following *Procedure* **a** or **b.**

Procedure a - The residue was purified by FCC to afford compounds **4-14.**

Procedure b - *Acetate deprotection procedure:* The residue was dissolved in methanol (5.0 mL). Then, NaOH (27 mg, 1.5 mmol) and water (2.0 mL) were added, and the mixture was stirred at 25 ° C for 6 hours. After evaporation of the solvent under vacuum (RV), diethyl ether was added. The solid formed was filtered, washed whit diethyl ether and purified by FCC affording compounds **15-20.**

Starting material, product, yield, chromatographic eluent, physical and analytical data of compounds **4-20** are as follows.

N-[(2-methyl-1H-indol-3-yl)(pyridin-2-yl)methyl]-3-nitroaniline (4)

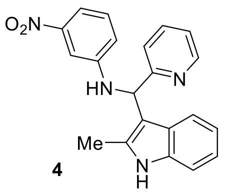

4

3-nitroaniline **1a** (119 mg); picolinaldehyde **2a** (107 mg); 2-methyl-1H-indole **3a** (125 mg); *purification:* procedure **a**, FCC-AcOEt/hexane (1:3). **4** (312 mg, 87%); ^{1}H NMR (300 MHz, CD_2Cl_2) δ 8.66 (d, 1H, *J* = 4.3 Hz), 8.20 (bs, 1H), 7.86 – 7.57 (m, 2H), 7.52 (s, 1H), 7.45 (d, 1H, *J* = 8.1 Hz), 7.36 – 7.21 (m, 4H), 7.04 – 6.99 (m, 3H), 6.66 (bs, 1H), 5.93 (s, 1H), 2.64 (s, 3H); ^{13}C NMR (75 MHz, CD_2Cl_2) δ 159.7, 149.3, 148.3, 148.2, 136.8, 135.5, 133.1, 129.4, 126.6, 122.2, 121.9, 121.3, 119.5, 118.7 (2 CH_{ar}), 111.3, 111.1, 110.5, 106.8, 54.6, 13.2. IR ν_{max} 3419, 3385, 3044, 1566, 1540, 1165, 832, 777 cm^{-1}. Anal. Calcd. for $C_{21}H_{18}N_4O_2$: C, 70.38; H, 5.06; N, 15.63. Found: C, 70.26; H, 4,98; N, 15.74.

***N*-[(2-methyl-1H-indol-3-yl)(pyridin-2-yl)methyl]-4-nitroaniline (5)**

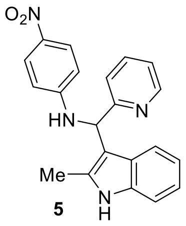

5

4-nitroaniline **1b** (119 mg); picolinaldehyde **2a** (107 mg); 2-methyl-1H-indole **3a** (125 mg); *purification:* procedure **a**, FCC-AcOEt/hexane (1:3). **5** (319 mg, 89%); ^{1}H NMR (300 MHz, CD_2Cl_2) δ 8.59 (d, 1H, *J* = 7.4 Hz), 8.27 (bs, 1H), 7.71 – 7.54 (m, 3H), 7.43 – 7.39 (m, 3H), 7.03 – 6.97 (m, 5H), 5.99 (bs, 1H), 5.95 (s, 1H), 2.68 (s, 3H); ^{13}C NMR (75 MHz, CD_2Cl_2) δ 160.1, 150.7, 149.3, 145.5, 145.3, 143.5, 140.9, 131.7, 130.4, 129.4, 126.6, (2 CH_{ar}), 126.3, 122.2, 121.3, 114.8 (2 CH_{ar}), 110.2, 108.6, 52.6, 12.9. IR ν_{max} 3419, 3385, 3044, 1566, 1540, 1165, 832, 777 cm^{-1}. Anal. Calcd. for $C_{21}H_{18}N_4O_2$: C, 70.38; H, 5.06; N, 15.63. Found: C, 70.26; H, 4,98; N, 15.77.

3-{[(2-methyl-1H-indol-3-yl)(pyridin-2-yl)methyl]amino}phenol (6)

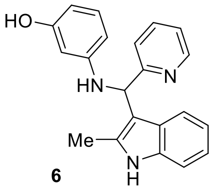

6

3-aminophenol **1c** (104 mg); picolinaldehyde **2a** (107 mg); 2-methyl-1H-indole **3a** (125 mg); *purification:* procedure **a**, FCC-AcOEt/hexane (1:2). **6** (204 mg, 62%); ^{1}H NMR (300 MHz, CD_3OD) δ 8.37 (d, 1H, *J* = 4.6 Hz), 7.65 (t, 1H, *J* = 7.8 Hz), 7.24-7.19 (m, 3H), 6.93 – 6.88 (m, 1H), 6.85 (d, 1H, *J* = 8.2 Hz), 6.75-6.64 (m, 2H), 6.30 (s, 1H), 6.25-6.14 (m, 2H), 6.01 (s, 1H), 2.14 (s, 3H) *NH and OH protons not visible*; ^{13}C NMR (75 MHz, CD_3OD) δ 164.6, 155.6, 147.6, 146.7, 135.6, 132.4, 130.4, 128.6, 123.4, 121.0, 119.6, 119.3,

118.2, 117.9, 111.6, 109.9, 107.0, 104.0, 102.7, 43.7, 10.7. IR ν $_{max}$ 3561, 3403, 3371, 3003, 1590, 1578, 1329, 1199, 901, 740 cm^{-1}. Anal. Calcd. for $C_{21}H_{19}N_3O$: C, 76.57; H, 5.81; N, 12.76. Found: C, 76.80; H, 5.91; N, 12.63.

N-[(5-fluoro-2-methyl-1H-indol-3-yl)(pyridin-2-yl)methyl]-3-nitroaniline (7)

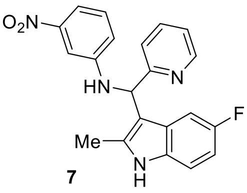

7

3-nitroaniline **1a** (119 mg); picolinaldehyde **2a** (107 mg); 5-fluoro-2-methyl-1H-indole **3b** (149 mg); *purification:* procedure **a**, FCC-AcOEt/hexane (1:3). **7** (309 mg 82%); ^{1}H NMR (300 MHz, CD_2Cl_2) *δ* 8.62 (d, 1H, *J* = 4.2 Hz), 8.22 – 7.99 (m, 2H), 7.65 – 7.58 (m, 2H), 7.46 – 7.43 (m, 1H), 7.31 – 7.29 (m, 1H), 7.24 – 7.12 (m, 4H), 6.81 – 6.67 (m, 1H), 6.41 (bs, 1H), 5.83 (s, 1H), 2.67 (s, 3H); ^{13}C NMR (75 MHz, CD_2Cl_2) *δ* 160.7, 157.0 (d, *J* = 252.1 Hz), 149.0, 148.8, 145.5, 136.4 (d, *J* = 2.1 Hz), 136.3, 134.8, 131.1, 126.7 (d, *J* = 7.4 Hz), 122.5 (2 CH_{ar}), 121.2, 117.2, 116.5, 116.2, 112.8 (d, *J* = 20.0 Hz), 110.7 (d, *J* = 8.1 Hz), 106.7 (d, *J* = 19.8 Hz), 53.0, 10.7. IR ν $_{max}$ 3420, 3400, 3101, 1593, 1521, 1296, 1219, 901, 763 cm^{-1}. Anal. Calcd. for $C_{21}H_{17}FN_4O_2$: C, 67.01; H, 4.55; N, 14.89. Found: C, 67.17; H, 4.67; N, 14.70.

N-[(1H-indol-3-yl)(pyridin-2-yl)methyl]-3-nitroaniline (8)

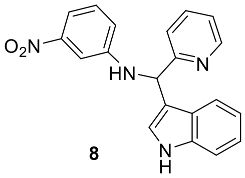

8

3-nitroaniline **1a** (131 mg); picolinaldehyde **2a** (107 mg); indole **3c** (117 mg); *purification procedure*: **method. a**, FCC-AcOEt/hexane (1:2). **8** (313 mg, 91%) ^{1}H NMR (300 MHz, CD_3OD) *δ* 8.56 (d, 1H, *J* = 3.8 Hz), 7.84 (t, 1H, *J* = 7.6 Hz), 7.69 (d, 1H, *J* = 7.5 Hz), 7.59 (d, 1H, *J* = 7.7 Hz), 7.48 (s, 1H), 7.43 (d, 1H, *J* = 7.8 Hz), 7.37 – 7.33 (m, 2H), 7.26 (t, 1H, *J* = 8.1 Hz), 7.13 (t, 1H, *J* = 7.5 Hz), 7.02 (t, 2H, *J* = 7.4 Hz), 6.92 (s, 1H), 6.02 (s, 1H) *OH and NH protons not visible*; ^{13}C NMR (75 MHz, CD_3OD) *δ* 157.6, 148.6, 148.3, 148.2, 136.8, 135.5, 133.1, 129.7, 126.6, 123.9, 123.1, 121.7, 121.3, 120.0, 118.8, 111.4, 111.12, 110.9, 106.5, 54.4. IR ν $_{max}$ 3419, 3385, 3044, 1566, 1540, 1165, 832, 777 cm^{-1}. Anal. Calcd. for $C_{20}H_{16}N_4O_2$: C, 69.76; H, 4.68; N, 16.27. Found: C, 69.60; H, 4.58; N, 16.39.

methyl 2-methyl-3-{[(3-nitrophenyl)amino](pyridin-2-yl)methyl}-1H-indole-6-carboxylate (9)

3-nitroaniline **1a** (119 mg); picolinaldehyde **2a** (107 mg); methyl 2-methyl-1H-indole-6-carboxylate **3d** (180 mg); *purification procedure*: **method. a**,

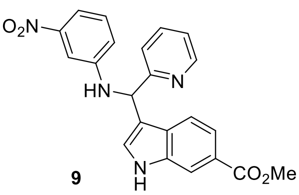

9

FCC-AcOEt/hexane (1:3). **9** (290 mg 76%); ^{1}H NMR (300 MHz, $CDCl_3$) δ 8.63 (d, 1H, *J* = 4.0 Hz), 8.47 (bs, 1H), 8.10 (s, 1H), 7.77 (d, 1H, *J* = 7.2 Hz), 7.67 – 7.60 (m, 2H), 7.51-7.46 (m, 3H), 7.28 – 7.18 (m, 3H), 6.94 (d, 1H, *J* = 7.2 Hz), 5.95 (bs, 1H), 5.94 (s, 1H), 3.91 (s, 3H); ^{13}C NMR (75 MHz, $CDCl_3$) δ 168.1, 160.1, 149.9, 149.8, 148.5, 137.7, 136.7, 130.3, 129.9, 127.0, 125.1, 123.3, 122.6, 121.8, 120.1, 119.6, 117.8, 114.5, 112.8, 108.1, 56.5, 52.7. IR ν_{max} 3323, 3301, 1772, 1543, 1302, 1200, 897, 700 cm^{-1}. Anal. Calcd. for $C_{22}H_{18}N_4O_4$: C, 65.66; H, 4.51; N, 13.92. Found: C, 65.78; H, 4.60; N, 13.80.

methyl 2-methyl-3-{[(3-nitrophenyl)amino](pyridin-2-yl)methyl}-1H-indole-5-carboxylate (10)

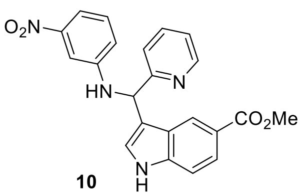

10

3-nitroaniline **1a** (119 mg); picolinaldehyde **2a** (107 mg); methyl 2-methyl-1H-indole-5-carboxylate **3e** (180 mg); *purification procedure*: **method. a**, FCC-AcOEt/hexane (1:3). **10** (275 mg 72%); ^{1}H NMR (300 MHz, CD_2Cl_2) δ 8.70 (d, 1H, *J* = 3.9 Hz), 8.68 (bs, 1H), 7.83- 7.77 (m, 2H), 7.70 – 7.40 (m, 5H), 7.21 – 7.00 (m, 4H), 6.01 (bs, 1H), 5.98 (s, 1H), 3.94 (s, 3H); ^{13}C NMR (75 MHz, $CDCl_3$) δ 165.4, 161.3, 150.1, 148.7, 148.6, 135.5, 135.1, 131.2, 129.9, 125.4, 124.9, 124.7, 124.6, 123.2, 122.2, 120.0, 117.5, 112.3, 110.7, 108.4, 57.3, 51.9. Anal. Calcd. for $C_{22}H_{18}N_4O_4$: C, 65.66; H, 4.51; N, 13.92. Found: C, 65.81; H, 4.62; N, 13.79.

***N*-[(6-fluoropyridin-2-yl)(2-methyl-1H-indol-3-yl)methyl]-3-nitroaniline (11)**

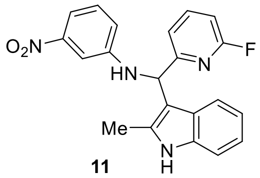

11

3-nitroaniline **1a** (119 mg); (6-fluoropyridin-2-yl)methanimine **2b** (124 mg); 2-methyl-1H-indole **3a** (125 mg); *purification:* procedure **a**, FCC-AcOEt/hexane (1:2). **11** (283 mg, 74%); ^{1}H NMR (300 MHz, $CDCl_3$) δ 9.65 (bs, 1H), 7.71 – 7.59 (m, 4H), 7.51-7.26 (m, 3H), 7.23 – 7.07 (m, 4H), 6.90 (dd, *J* = 7.7, 1.5 Hz, 1H), 5.91 (s, 1H), 2.62 (s, 3H); ^{13}C NMR (75 MHz, $CDCl_3$). δ 161.0 (d, *J* = 251.8 Hz), 150.0, 148.5, 147.3, 142.8 (d, *J* = 8.1 Hz), 142.1, 135. 9, 131.7, 130.1 (d, *J* = 7.9 Hz), 128.7, 125.0 (d, *J* = 3.1 Hz), 123.8, 123.0, 120.3, 119.4, 117.1, 111.9, 111.4, 106.2 (d, *J* = 19.8 Hz), 55.7, 11.9. IR ν_{max} 3420, 3400, 3101, 1593, 1521, 1296, 1219, 901, 763 cm^{-1}. Anal. Calcd. for $C_{21}H_{17}FN_4O_2$: C, 67.01; H, 4.55; N, 14.89. Found: C, 67.15; H, 4.64; N, 14.75.

2-{(2-methyl-1H-indol-3-yl)[(3-nitrophenyl)amino]methyl}pyridin-3-ol (12)

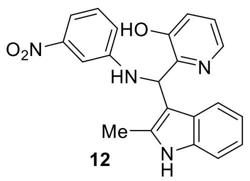

12

3-nitroaniline **1a** (119 mg); 2-(iminomethyl)pyridin-3-yl acetate **2c** (164 mg); 2-methyl-1H-indole **3a** (125 mg); *purification:* procedure **b**, FCC-AcOEt/hexane (1:2). **12** (191 mg, 51%); ^{1}H NMR (300 MHz, Acetone-d_6) δ 10.21 (bs, 1H), 9.46 (bs, 1H), 8.52 (d, 1H, J = 4.6 Hz), 8.24 (d, 1H, J = 7.6 Hz), 7.93 (s, 1H), 7.66 – 7.39 (m, 6H), 7.30 – 7.17 (m, 2H), 6.64 (bs, 1H), 6.63 (s, 1H), 3.10 (s, 3H); ^{13}C NMR (75 MHz, Acetone-d_6) δ 150.6, 149.6, 149.0, 147.9, 139.5, 136.2, 134.1, 129.7, 127.5, 123.2, 122.5, 120.5 (2 CH_{ar}), 120.0, 118.8, 110.5, 110.4, 109.5, 106.4, 49.6, 11.8. IR ν_{max} 3422, 3417, 3309, 3060, 1561, 1530, 1324, 876, 760 cm^{-1}. Anal. Calcd. for $C_{21}H_{18}N_4O_3$: C, 67.37; H, 4.85; N, 14.96. Found: C, 67.16; H, 4.72; N, 15.08

N-[(2-methyl-1H-indol-3-yl)(phenyl)methyl]-3-nitroaniline (13)

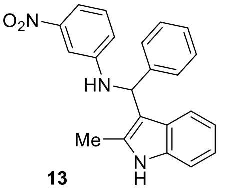

13

3-nitroaniline **1a** (119 mg); benzaldehyde **2d** (106 mg); 2-methyl-1H-indole **3a** (125 mg); *purification*: procedure **a**, FCC-AcOEt/hexane (1:3). **13** (314 mg, 88%). ^{1}H NMR (300 MHz, CD_2Cl_2) δ 8.42 – 7.99 (m, 1H), 7.89 – 7.64 (m, 2H), 7.58 (t, J = 7.5 Hz, 1H), 7.47 – 7.37 (m, 3H), 7.37 – 7.27 (m, 4H), 7.15 – 7.03 (m, 2H), 6.87 – 6.79 (m, 1H), 6.77 (bs, 1H), 6.74 (s, 1H), 2.34 (s, 3H); ^{13}C NMR (75 MHz, CD_2Cl_2) δ 159.7, 149.3, 148.3, 148.2, 136.8, 135.5, 133.1, 129.4, 126.6, 122.2, 121.9, 121.3, 119.5 (2 CH_{ar}), 118.7 (2 CH_{ar}), 111.3, 111.1, 110.5, 106.8, 54.6, 12.7. IR ν_{max} 3391, 3244, 1600, 1551, 1186, 802, 788 cm^{-1}. Anal. Calcd. for $C_{22}H_{19}N_3O_2$: C, 73.93; H, 5.36; N, 11.76. Found: C, 73.80; H, 5.31; N, 11.85.

N-[(5-fluoro-2-methoxyphenyl)(2-methyl-1H-indol-3-yl)methyl]-3-nitroaniline (14)

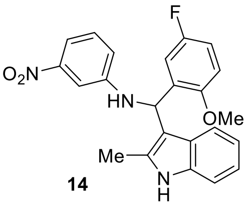

14

3-nitroaniline **1a** (119 mg); 5-fluoro-2-methoxybenzaldehyde **2e** (154 mg); 2-methyl-1H-indole **3a** (125 mg); *purification* procedure **a**, FCC-AcOEt/hexane (1:2). **14** (289 mg, 75%); ^{1}H NMR (400 MHz, Acetone- d_6) δ 10.06 (bs, 1H), 7.62 – 7.53 (m, 2H), 7.49 (dd, J = 9.6, 2.9 Hz, 1H), 7.44 (dd, J = 8.0, 2.2 Hz, 1H), 7.33 (d, J = 8.1 Hz, 1H), 7.31 – 7.23 (m, 1H), 7.07 (dd, J = 8.1, 2.4 Hz, 1H), 7.03 – 6.97 (m, 3H), 6.96 – 6.88 (m, 1H), 6.22 (d, J = 4.5 Hz, 1H), 6.16 (d, J = 4.4 Hz, 1H), 3.84 (s, 3H), 2.52 (s, 3H); ^{13}C NMR (75 MHz, Acetone-d_6) δ 158.2, 154.6 (d, J = 248.7 Hz), 149.3, 149.2, 135.70, 133.1, 132.2 (d, J = 4.9 Hz), 129.6, 127.1, 120.4, 118.9, 118.8, 118.7, 114.0 (d, J = 24.9 Hz), 113.6 (d, J = 22.9 Hz), 112.2 (d, J = 8.1 Hz), 110.7, 110.5, 109.9, 106.7, 55.6, 49.5, 11.3; IR ν_{max} 3385,

3334, 1735, 1500, 1309, 1201, 899 cm^{-1}. Anal. Calcd. for $C_{23}H_{20}FN_3O_3$: C, 68.14; H, 4.97; N, 10.36. Found: C, 68.25; H, 5.04; N, 10.27

2-{(2-methyl-1H-indol-3-yl)[(3-nitrophenyl)amino]methyl}phenol (15)

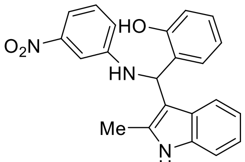

15

3-nitroaniline **1a** (119 mg); 2-(iminomethyl)phenyl acetate **2f** (163 mg); 2-methyl-1H-indole **3a** (125 mg); *purification:* procedure **b**, FCC-AcOEt/hexane (1:2). **15** (229 mg, 63%); ^{1}H NMR (300 MHz, DMSO-d_6) δ 10.80 (bs, 1H), 9.48 (bs, 1H), 7.43 (d, 1H, *J* = 7.8 Hz), 7.39 (d, 1H, *J* = 7.5 Hz), 7.34 (s, 1H), 7.25 – 7.17 (m, 3H), 7.00 (t, 1H, *J* = 7.4 Hz), 6.92 – 6.87 (m, 3H), 6.82 -6.77 (m, 2H), 6.70 (t, 1H, *J* = 7.4 Hz), 5.98 (d, 1H, *J* = 5.3 Hz), 2.32 (s, 3H). ^{13}C NMR (75 MHz, DMSO-d_6) δ 155.1, 149.7, 149.0, 135.5, 133.2, 130.0, 128.4, 128.1, 128.0 (2 CH_{ar}), 127.5, 120.3, 119.1, 119.0, 118.7, 115.7, 110.9, 110.4, 109.9, 106.2, 48.7, 12.3. IR ν_{max} 3475, 3449, 3399, 1588, 1531, 1354, 862, 750 cm^{-1}. Anal. Calcd. for $C_{22}H_{19}N_3O_3$: C, 70.76; H, 5.13; N, 11.25. Found: C, 70.91; H, 5.21; N, 11.14.

3-{(2-methyl-1H-indol-3-yl)[(3-nitrophenyl)amino]methyl}phenol (16)

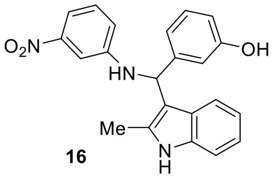

3-nitroaniline **1a** (119 mg); 3-(iminomethyl) phenyl acetate **2g** (164 mg); 2-methyl-1H-indole **3a** (125 mg); *purification:* procedure **b**, FCC-AcOEt/hexane (1:2). **16** (256 mg, 72%); ^{1}H NMR (400 MHz, CD_3OD) δ 7.44 (t, 1H, *J* = 2.3 Hz), 7.41 (d, 1H, *J* = 8.0 Hz), 7.37 (dd, 1H, *J* = 8.1, 2.2 Hz), 7.27 – 7.13 (m, 3H), 7.01 – 6.88 (m, 4H), 6.75 – 6.65 (m, 2H), 5.81 (s, 1H), 2.39 (s, 3H) *OH and NH protons not visible*; ^{13}C NMR (101 MHz, CD_3OD) δ 157.1, 149.4, 149.1, 146.3, 144.0, 132.6, 129.0, 127.2, 120.1, 118.7, 118.3, 118.2, 117.8, 113.8, 113.4, 112.5, 110.1, 110.0, 109.7, 106.3, 54.6, 10.7. IR ν_{max} 3477, 3466, 3415, 1579, 1525, 1361, 890, 724 cm^{-1}. Anal. Calcd. for $C_{22}H_{19}N_3O_3$: 70.76; H, 5.13; N, 11.25. Found: C, 70.52; H, 5.01; N, 11.43.

3-{(2-methyl-1H-indol-3-yl)[(3-nitrophenyl)amino]methyl}phenol (17)

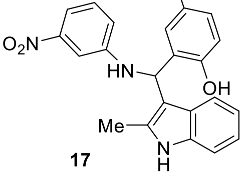

17

3-nitroaniline **1a** (119 mg); 4-fluoro-2-(iminomethyl)phenyl acetate **2h** (181 mg); 2-methyl-1H-indole **3a** (125 mg); *purification:* procedure **b**, FCC-AcOEt/hexane (1:2). **17** (234 mg, 63%); ^{1}H NMR (300 MHz, Acetone- d_6) δ

10.05 (bs, 1H), 9.86 (bs, 1H), 7.57 (d, *J* = 3.6 Hz, 1H), 7.44 (d, *J* = 8.3 Hz, 1H), 7.35 – 7.25 (m, 3H), 7.12 (d, 1H, *J* = 8.1 Hz), 6.82 – 6.71 (m, 4H), 6.75 (t, *J* = 7.6 Hz, 1H), 6.24 (bs, 1H), 6.20 (s, 1H), 2.38 (s, 3H); ^{13}C NMR (75 MHz, Acetone-d_6) δ 155.0, 153.2 (d, *J* = 260.3 Hz), 151.1, 149.3, 135.7 (d, *J* = 10.9 Hz), 133.2, 132.1, 129.6, 129.0, 127.2, 120.4, 119.9, 119.3 – 118.5 (m, 3CH_{ar}), 118.3, 110.8, 110.5, 110.2, 106.8, 49.9, 11.3. IR ν_{max} 3508, 3407, 3334, 1561, 1532, 1300, 1201, 867, 788 cm^{-1}. Anal. Calcd. for $C_{22}H_{18}FN_3O_3$: C, 67.51; H, 4.64; N, 10.74. Found: C, 67.36; H, 4.54; N, 10.85

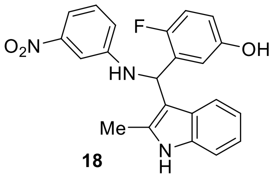

4-fluoro-3-{(2-methyl-1H-indol-3-yl)[(3-nitrophenyl)amino]methyl}phenol (18)

3-nitroaniline **1a** (119 mg); 4-fluoro-3-(iminomethyl)phenyl acetate **2i** (181 mg); 2-methyl-1H-indole **3a** (125 mg); *purification:* procedure **b**, FCC-AcOEt/hexane (1:2). **18** (270 mg, 70%). ^{1}H NMR (400 MHz, CD_3OD) δ 7.89 (s, 1H), 7.50 (d, 1H, *J* = 7.5 Hz), 7.31 (s, 1H), 7.29 – 7.05 (m, 4H), 6.95 – 6.81 (m, 2H), 6.68 – 6.57 (m, 2H), 5.90 (s, 1H), 2.48 (s, 3H) *OH and NH protons not visible*; ^{13}C NMR (101 MHz, CD_3OD) δ 156.7 (d, *J* = 252.1 Hz), 153.9, 149.5, 145.8, 139.1, 137.21, 131.8, 129.3 (d, *J* = 20.0 Hz), 128.4, 123.3, 123.1, 121.7 (d, *J* = 1.9 Hz), 120.0, 119.4 (2CH_{ar}), 116.7 (d, *J* = 20.0 Hz), 116.4 (d, *J* = 8.1 Hz), 116.0 (d, *J* = 8.1 Hz), 111.5, 111.4, 50.1, 12.2. IR ν_{max} 3508, 3407, 3334, 1561, 1532, 1300, 1201, 867, 788 cm^{-1}. Anal. Calcd. for $C_{22}H_{18}FN_3O_3$: C, 67.51; H, 4.64; N, 10.74. Found: C, 67.66; H, 4.75; N, 10.61.

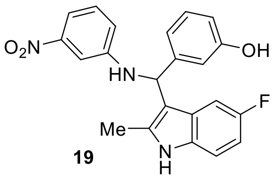

3-{(5-fluoro-2-methyl-1H-indol-3-yl)[(3-nitrophenyl)amino]methyl}phenol (19)

3-nitroaniline **1a** (119 mg); 3-(iminomethyl)phenyl acetate **2g** (163 mg); 5-fluoro-2-methyl-1H-indole **3b** (149 mg); *purification:* procedure **b**, FCC-AcOEt/hexane (1:2). **19** (278 mg, 71%); ^{1}H NMR (400 MHz, $CDCl_3$) δ 7.93 (bs, 1H), 7.53 – 7.51 (m, 2H), 7.34 (s, 1H), 7.25 – 7.02 (m, 5H), 6.87 – 6.77 (m, 2), 6.69 – 6.62 (m, 1H), 5.93 (s, 1H), 5.05 (bs, 1H), 6.90 (bs, 1H), 2.45 (s, 3H); ^{13}C NMR (101 MHz, $CDCl_3$) δ 156.9, 156.1 (d, *J* = 247.8 Hz), 146.6, 145.9, 145.0, 136.2, 135.0 (d, *J* = 2.8 Hz), 130.0, 129.3, 124.9 (d, *J* = 7.9 Hz), 123.8, 119.6, 119.0 (d, *J* = 3.1 Hz), 116.2, 115.9, 115.0(d, *J* = 20.0 Hz), 114.5, 111.2, 110.3 (d, *J* = 8.1 Hz), 107.9 (d, *J* = 20.0 Hz), 54.8, 13.0. IR ν_{max} 3508, 3407, 3334, 1561, 1532, 1300, 1201, 867, 788 cm^{-1}. Anal. Calcd. for $C_{22}H_{18}FN_3O_3$: C, 67.51; H, 4.64; N, 10.74. Found: C, 67.78; H, 4.79; N, 10.51.

4-fluoro-3-{(5-fluoro-2-methyl-1H-indol-3-yl)[(3-nitrophenyl)amino]methyl}phenol (20)

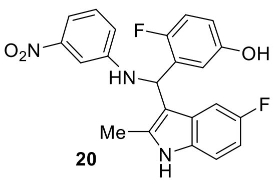

20

3-nitroaniline **1a** (119 mg); 4-fluoro-3-(iminomethyl)phenyl acetate **2h** (181 mg); 5-fluoro-2-methyl-1H-indole **3b** (149 mg); *purification:* procedur **b**, FCC-AcOEt/hexane (1:2). **20** (305 mg, 73%); ^{1}H NMR (400 MHz, $CDCl_3$) δ 7.78 (bs, 1H), 7.59 – 7.48 (m, 2H), 7.27 – 7.02 (m, 4H), 6.95 – 6.68 (m, 3H), 6.53 – 6.50 (m, 2), 5.96 (s, 1H), 2.15 (s, 3H) *OH proton not visible*; ^{13}C NMR (101 MHz, $CDCl_3$) δ 159.4, 157.7 (d, J = 238.9 Hz), 150.2, 147.4, 145.8, 135.6, 134.1 (d, J = 2.7 Hz), 129.5, 129.4, 128.6 (d, J = 7.9 Hz), 122.7, 117.8 (d, J = 3.1 Hz), 117.5, 116.1, 116.0, 114.6, 114.4 (d, J = 21.0 Hz), 111.5 (d, J = 8.1 Hz), 111.2, 106.7 (d, J = 20.0 Hz), 51.7, 13.1. IR ν_{max} 3508, 3407, 3334, 1561, 1532, 1300, 1201, 867, 788 cm^{-1}. Anal. Calcd. for $C_{22}H_{17}F_2N_3O_3$: C, 64.54; H, 4.19; N, 10.26. Found: C, 64.78; H, 4.33; N, 10.12.

Biological evaluation

In vitro kinase assays were performed using the EGFR(T790M/L858R) Kinase Assay Kit (BPS Bioscience, Cat. #40322) in the presence of compounds or vehicle. Assays conditions were set according to the manufacturer's instructions. Dose-response curves were fitted by non-linear regression of normalized enzyme activity *vs* log[inhibitor] data, using GraphPad Prism software. The IC_{50} value is defined as the concentration of compound that inhibits 50% enzyme activity. Compound **4** and **EAI045** were used as reference compounds in all experiments.

For cytotoxicity assays, compounds were added to cells in 96-well plates for 72 hours. Cell viability was measured by MTS assay using the CellTiter 96® AQueous One Solution Cell Proliferation Assay System (Promega, Madison, WI, USA) according to instructions. Inhibition of EGFR was assessed by Western blotting after incubating the cells with the compounds for 8 hours. Cells were then harvested, lysed and equal total protein lysates were loaded on SDS-PAGE gels and probed with phospho-EGFR (Tyr1068), total EGFR and actin specific antibodies (Cell Signaling). Chemiluminescence was detected using a ChemiDoc XRS+ System (Bio-Rad, Hercules, CA, USA) and Image Lab software (Bio-Rad).

Molecular docking analyses

All molecules were drawn by using Maestro of the Schrödinger 2021-1 suite[1]. Then, the *LigPrep* tool was used to generate all possible protomers and tautomers potentially present in the pH range of 6-8, and to obtain minimized structures suitable for molecular docking.

The PDB structure of mutant EGFR complexed with EAI001 (PDB ID: 5D41)[2] was downloaded from the Protein Data Bank website. The chain B of the downloaded PDB complex was deleted, as it has not been co-crystallized with EAI001 allosteric ligand.[2] Chain A was processed by using the *Protein Preparation Wizard* toolkit[3] to fix atom types and bond connectivity issues, to rebuild missing residue side chains and to add hydrogen atoms. Finally, a restrained minimization was performed on the structure according to the OPLS4 force field, to relieve steric clashes, until a final root means square deviation (RMSD) of 0.3 Å, with respect to the input protein coordinates. Afterwards, all water molecules were removed from the minimized complex, except one (residue number 1285) which was previously investigated for its importance in the binding mode of EAI001 and EAI045[4].

A receptor grid was then built on the prepared structure by centering the enclosing box (default dimensions of 10 Å × 10 Å × 10 Å) at the centroid of the workspace ligand. Molecular docking calculations were performed by using the Extra Precision (XP) mode of the *Glide* software (Schrödinger 2021-1)[5]. The protocol reported in our previously published work was applied by using the same settings, with enhancement of the conformational space of ligand sampling by a factor of 4, and 50 poses per ligand submitted to post-docking minimization[6]. All other parameters were set to their default values.

To account for binding site flexibility, additional docking experiments were also performed with the *Induced Fit Docking* (IFD) protocol (Schrödinger Suite 2021-1)[7]. IFD calculations were conducted with the standard settings. All poses were visually inspected and manually clustered according to the obtained binding modes.

Figure S1: Dose-response curves of compounds **15** (panel A) and **18** (panel B) against H1975 (Lung adenocarcinoma, double mutant EGFR), H3122 (Lung adenocarcinoma, wild type EGFR) and HL60 (Acute myeloid leukemia, negative EGFR) cell lines.

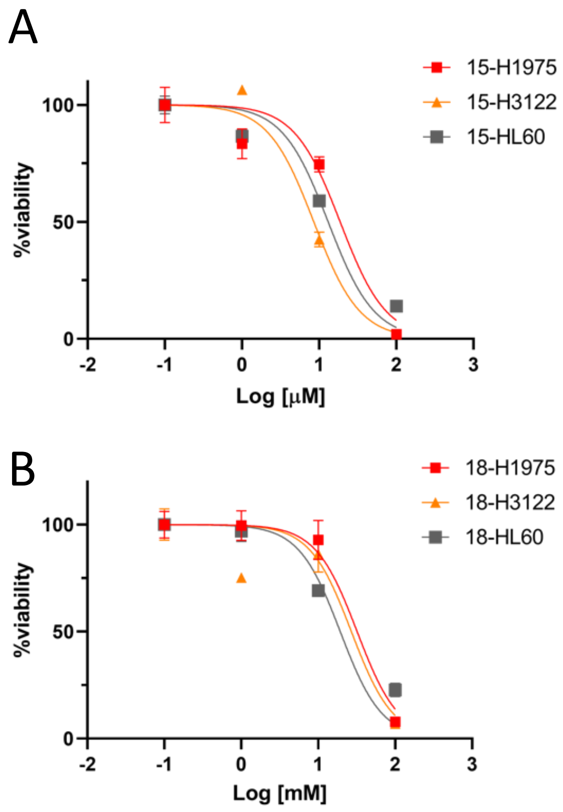

Table S1: *In vitro* inhibitory activity of the most relevant compounds obtained through the EGFR(T790M/L858R) Kinase Assay Kit. Compounds **4** and **EAI045** were used as references.

Compound ID	***IC50 (µM)* ± *Std. Dev.***
**EAI045**	9 ± 2
**4**	154 ± 11
**7**	123 ± 17
**8**	95 ± 31
**12**	96 ± 30
**15**	33 ± 4
**17**	63 ± 12
**18**	27 ± 5
**20**	200 ± 142

REFERENCES

1. Schrödinger Release 2021-1: Maestro, Schrödinger, LLC, New York, NY, 2021.
2. Jia Y, Yun C-H, Park E, et al. Overcoming EGFR T790M and C797S Resistance with Mutant-Selective Allosteric Inhibitors. Nature 2016; 534:129–132.
3. Schrödinger Release 2021-2: Protein Preparation Wizard; Epik, Schrödinger, LLC, New York, NY, 2021; Impact, Schrödinger, LLC, New York, NY; Prime, Schrödinger, LLC, New York, NY, 2021.
4. Tinivella A, Rastelli G. Investigating the Selectivity of Allosteric Inhibitors for Mutant T790M EGFR over Wild Type Using Molecular Dynamics and Binding Free Energy Calculations. ACS Omega 2018; 3(12):16556–16562.
5. Friesner RA, Banks JL, Murphy RB, et al. Glide: a new approach for rapid, accurate docking and scoring. 1. Method and assessment of docking accuracy. J Med Chem 2004; 47:1739–49.
6. Caporuscio F, Tinivella A, Restelli V, et al. Identification of small-molecule EGFR allosteric inhibitors by high-throughput docking. Future Med. Chem. 2018; 10(13):1545-1553.
7. Schrödinger Release 2021-1: Induced Fit Docking protocol; Glide, Schrödinger, LLC, New York, NY, 2021; Prime, Schrödinger, LLC, New York, NY, 2021.
